# How to evaluate user experience in digital health? A scoping review of questionnaires in virtual reality applications

**DOI:** 10.3389/fdgth.2025.1561364

**Published:** 2025-09-15

**Authors:** Francesca Bruni, Valentina Mancuso, Elisa Pedroli

**Affiliations:** ^1^ Department of Theoretical and Applied Sciences, eCampus University, Novedrate, Italy; ^2^ Older People Rehabilitation and Cerebrovascular Medicine Research Laboratory, IRCCS Istituto Auxologico Italiano, Milan, Italy

**Keywords:** user experience, virtual reality, aging, health, questionnaire

## Abstract

The exponential growth and integration of virtual reality technology in clinical environments necessitates a comprehensive user experience evaluation. This assessment is critical for clinical populations and geriatric cohorts presenting peculiar needs and expectations. Despite the longstanding conceptual framework of user experience, a consensus regarding its definition and optimal evaluation methodologies remains elusive, especially within healthcare contexts. This systematic scoping review examines state-of-the-art questionnaire-based instruments for assessing user experience in healthcare virtual reality applications, synthesizing current evaluation approaches and identifying key user experience dimensions. Following the Preferred Reporting Items for Systematic Reviews and Meta-Analyses for Scoping Review guidelines, we analyzed articles employing questionnaires to evaluate user experience in virtual reality-based health applications for adults. Following a meticulous screening process of 325 papers across PubMed, Web of Science, and Embase databases, 17 studies met our inclusion criteria. Studies predominantly used multiple and diverse questionnaires exploring several dimensions. Eight key user experience dimensions emerged: usability and functionality, aesthetics of design, engagement, emotional state, presence, realism of environments, side effects, and motivation and intention of use. Current evaluation lacks standardization and theoretical consistency. We propose a comprehensive eight-domain framework and recommend integrating multidisciplinary expertise, implementing longitudinal evaluation approaches, and developing psychometrically validated instruments. These findings provide essential guidance for improving patient outcomes and healthcare delivery efficacy through optimized virtual reality-based implementation.

## 1 Introduction

The rapid advancement of novel technologies has led to the proliferation of innovative instruments in clinical domains. In the field of neuropsychology, in particular, there is a growing interest in designing cutting-edge tools for both clinical and research applications, as well as the expansion of studies on their efficacy in assessing and training patients (1, 2). Among these emerging tools Virtual Reality (VR) has garnered significant attention for its potential to transform healthcare delivery and patients outcomes. However, the successful implementation of VR depends critically on understanding and optimizing the User Experience (UX). Despite the importance of evaluating UX and its long-standing conceptual history, there is still no consensus regarding the definition and optimal research methodologies for UX. This ambiguity is particularly pronounced in healthcare contexts, where the stakes of technology adoption are high and user populations present unique characteristics and needs Some authors have attributed the absence of a commonly accepted definition to the presence of a broad and fuzzy range of variables associated with UX (3), often attributable to the author's background and interest. Moreover, the UX unit of analysis is too changeable, ranging from a single aspect of an individual user's contact with an application to all aspects of many users’ interactions with a company and the integration of multiple disciplines and services. Thus, the UX field is fragmented and confused by various theoretical models with diverse foci such as device features, emotion, affect, value, enjoyment, and beauty (3). This complexity becomes even more pronounced when considering the specific requirements of healthcare applications, where VR technologies must not only provide engaging experiences but also ensure safety, accessibility, and therapeutic efficacy across diverse clinical populations. The convergence of VR technology, UX principles, and healthcare applications represents a critical yet understudied intersection that demands systematic investigation and standardization.

This review is structured to provide a comprehensive examination of UX evaluation questionnaire-based in VR healthcare applications. Following this introduction, which establishes the theoretical foundations of UX and its specific relevance to VR healthcare applications, the methodology section details the systematic scoping review approach, and results section presents findings on participant populations, VR application characteristics, and UX evaluation instruments, culminating in modelling eight key UX domains. Finally, we propose recommendations for future research and practice, while acknowledging the study's limitations and suggesting directions for potential advancement.

### 1.1 User experience

The term UX initially arose in the field of Human-Computer Interaction and technology design. A recent definition from ISO/IEC 30071-1:2019 broadly describes UX as “a person's perception and responses that result from the use or anticipated use of a product, system or service” (4, 5). Although it has been perfected over time, this definition provides only a general and unclear conceptualization, failing to delineate the range of factors underlying UX. Nonetheless, UX is not much different from experience *per se* (6), which partly explains the inherent difficulty in precisely defining it. Considering the interaction with technologies, the term experience encompasses all aspects of how people interact with a product, including the way it feels in their hands, comprehension of functionality, emotional responses during use, efficacy in purpose fulfillment, and contextual appropriateness (7). The complexity of defining UX stems from the fusion of all these multiple components, including users' internal states (emotions, expectations, and active goals), which persist and evolve. Despite this complexity, the core agreement is its dynamism and subjectivity (6, 8–10).

One approach in defining the concept of UX is to characterize specific dimensions of which it is composed. Among the various models proposed over time, a consensus has emerged among researchers that UX is composed of pragmatic and hedonic aspects (11). Pragmatics refers to instrumental qualities of a system, which relate to perceived usefulness, effectiveness, and ease of use. They are associated with the so-called utility and usability aspects. This dimension pertains to the achievement of specific goals, such as successfully sending a message on a mobile device. The hedonic attributes (non-instrumental) consider the “joy of use” highlighting the stimulation, identification, and evocation that arise from product utilization (12). In the context of the previous example, this would relate to the affective experience of being able to send the message. While pragmatic and hedonic are conceptualized as distinct aspects, these characteristics are strongly positively correlated (13). They work in tandem to elicit either positive or negative feelings, which guides the success of the experience (14, 15). However, this distinction provides a somewhat limited view of UX. Numerous other factors play significant roles in shaping UX, and these factors have become increasingly focal points of research over time.

Beyond the pragmatic-hedonic dichotomy, contemporary UX theory recognizes the critical role of user motivation and intentions in shaping technological experiences (16). Users approach systems with varying intrinsic and extrinsic motivations that fundamentally influence their perception and evaluation of the interaction. Intrinsic motivation, driven by personal satisfaction and enjoyment, often enhances hedonic aspects of UX, while extrinsic motivation, focused on achieving specific outcomes, primarily influences pragmatic dimensions. Furthermore, users' intentions—whether exploratory, task-oriented, or social—create different expectation frameworks that directly impact their subjective experience (17). This motivational framework becomes particularly relevant in healthcare contexts, where patients may interact with VR systems driven by therapeutic goals, curiosity, or compliance with medical recommendations, each creating distinct UX patterns. Contextual appropriateness emerges as another fundamental dimension of UX theory, extending beyond the immediate user-system interaction to encompass the broader situational, cultural, and environmental factors that influence experience quality. Contextual factors include the physical setting (e.g., clinical environment, home setting), social context (e.g., presence of healthcare professionals, family members), temporal constraints (e.g., time pressure, treatment duration), and cultural considerations (e.g., technology acceptance within specific populations), considering how well a system aligns with the specific context of use (18). In healthcare applications, contextual appropriateness becomes critically important as technologies must seamlessly integrate into clinical workflows, accommodate diverse patient populations, and respect institutional protocols while maintaining therapeutic efficacy. This contextual dimension is closely related to the system's purpose and objective. Van der Heijden (19) noted the most important variables influencing UX fluctuate according to the system's goal and purpose, especially concerning hedonic aspects. Depending on the purpose of a system, usefulness may lose its dominant predictive value in favor of enjoyment. In certain instances, a product's emotion, aesthetic appeal, and capacity to reinforce user identity may play a major role in determining whether a user has a positive or bad experience. Conversely, more utilitarian products may rely more heavily on factors such as user engagement, perceived usefulness, and quality of interaction (19).

The recognition of these multidimensional aspects has led research to expand beyond instrumental qualities to include personal features of users as crucial aspects in shaping technological experiences. Gender and age, for example, present notable differences in technology experiences. Men and women exhibit distinct patterns in their decision-making processes regarding technology adoption, access, utilization, and perceived ease of use. These differences are so pronounced that literature talks about gender digital divide (20). Furthermore, older adults frequently perceive themselves as unable to interact with technologies such as smartphones or tablets. Their self-perceived inefficacy often hinders the adoption of these technological innovations. Notably, these systems are predominantly designed with a young public in mind, rendering interaction potentially complex and frustrating for aging (21). This is evidence of how users' reactions to technologies might be strongly influenced by personal, non-instrumental aspects. This evolution in understanding has helped distinguish UX from the narrower concept of usability, with which it was historically confused and intertwined. Someone still use the term UX to refer to usability in general, but this represents a limited perspective. Usability is an important factor, but it just provides a pragmatic overview confined to the objective point of view that does not address user satisfaction or the subjective experience of interacting with technology (22). Since 1996 Alben, for example, introduced aesthetics as an important aspect of technology (7), as also more recently highlighted by Lavie and Tractinsk (23) who demonstrated how the aesthetics of interfaces is a strong determinant of users' satisfaction and pleasure. This body of literature demonstrates that the pragmatic aspect is not enough; researchers need to pay attention to the hedonic dimensions. Moving away from the limitation of functional aspects a lot of non-instrumental features have been introduced over the years, further delineating the concept of UX and detaching from the device-related quality of the usability paradigm. This distinction between UX and usability becomes evident when considering the role of implementation context. While usability traditionally focuses on interface ease of use that can be analyzed in controlled laboratory setting, UX is particularly sensitive to context, as it involves subjective and dynamic elements encompassing a border analytic framework to include real-word environmental factors (24). An interface may demonstrate excellent usability in isolation, yet when implemented in actual contexts of use, its experience can be significantly affected by physical, social, and cultural factors that influence interaction. For instance, a system may function during controlled testing but face substantial barriers when deployed in a specific context such as clinical settings due to space constraints, workflow interruptions, or staff resistance—factors that only become apparent through comprehensive contextual framework. Among the vivid discussion surrounding the multifaceted and dynamic concept of UX, it becomes evident that the concept varies significantly depending on several factors, including product characteristics, user attributes, and contextual elements. However, it is imperative to recognize that time constitutes another crucial variable warranting consideration in this complex equation (25). UX is not a stable phenomenon since the user changes, the system changes, and the entire context of use changes, in an intense dynamism of factors. Indeed, UX evaluations must be dynamically conceived to resonate with specific user populations and their contemporary socio-technical contexts. As technologies evolve and societal needs transform, relying on outdated assumptions originally designed for different eras can lead to significant usability gaps and user friction.

### 1.2 UX in VR application for health

Virtual Reality (VR) refers to a technology that creates a three-dimensional environment with which users can interact through specialized hardware, engaging in a seemingly real or physical reality, despite the absence of physical interaction (26, 27). This technology emerges as a promising tool in healthcare applications, where the ability to create controlled, reproducible, and safe virtual environments offers unprecedented opportunities for medical training, patient treatment, and therapeutic interventions. These applications span a wide range of areas such as mood modulation (16), balance and gait improvement (17, 18), and limb function enhancement (28). Moreover, VR has shown potential in treating specific disorders such as eating disorders (29) and addressing aging-related conditions whether physiological or pathological conditions (30–33). In the context of clinical conditions, VR might help to enhance the quality of life, improve healthcare delivery, and reduce social costs throughout the lifespan (34, 35). Furthermore, VR applications can assist both patients and clinicians in supporting clinical decision-making processes, facilitating health record access, and enhancing communication (36). Additionally, VR aids patients in managing their health status through diet, exercise, and chronic diseases, while also improving interactions with caregivers (37), offering new possibilities for patient assessment and treatments. However, to fully harness the potential of this technology, it is crucial to carefully evaluate the UX. The concept is shaped by a multitude of factors, including personal characteristics, contextual variables, and the purpose of use. Thus, attention to the specific attributes of end-users and the nuances of their usage context is crucial to effectively delineate the concept and facilitate accurate assessment, as well as optimal design of technological instruments.

Evaluating UX during the design of VR-based applications for clinical use is crucial for several interconnected reasons that extend far beyond interface usability. The implementation context emerges as a critical factor. When deploying VR technologies in healthcare environments, numerous contextual variables must be considered that significantly impact UX but remain invisible in laboratory testing conditions. Hospital environments present unique spatial and infrastructure constraints such as room layouts, availability of adequate space, proximity to the essential medical equipment, power socket and Wi-Fi connectivity strength, noise level all may create unexpected barriers that compromise the intended user experience—affecting both VR performance and patient comfort during session-, influencing the feasibility of interventions (38, 39). Capturing these contextual factors requires UX evaluation methods specifically designed for real-world implementation analysis. Contextual inquiry allows researchers to observe and understand how VR systems perform within actual healthcare workflows, revealing implementation challenges that controlled testing cannot anticipate.

Beyond these implementation contexts, VR applications must also address user-specific factors that influence experience quality. Design errors can endanger patient safety, making accurate UX analysis essential to ensure applications are accessible and usable by diverse user populations. This is particularly evident in aging people who may have limited experience with technology or may feel uncomfortable using it (21). An intuitive and easy-to-use interface, for example, can reduce anxiety and increase patient confidence, leading to more accurate results and improving adherence to rehabilitation programs (37). However, these user-centered design considerations must be evaluated within the broader implementation context to ensure that solutions remain effective when deployed in real healthcare environments.

Several barriers can also hinder the use of VR technologies, including cognitive and physical limitations. Aging is associated with physiological changes that may lead to declines in sensory, mental, and physical functioning (35). Clinical conditions, such as neurological diseases or frailty, further threaten the well-being of older adults (40, 41). They may have difficulty in working memory, spatial cognition, attention, language, reasoning, motor speed, flexibility, hand-eye coordination, and strength, which makes using VR more challenging. Based on a pragmatic point of view, the application features must be sewn on these features characterized end-user. Quality of VR graphics (e.g., resolution, movement of visual elements, shapes, color contrast), synchronization (i.e., the delay between the users' movement into the VR environment and what it is expected to see), as well as user interface layouts, are elements that could be influenced by clinical conditions. While a playful interaction—composed of a great number of colors and sounds—might be crucial for the success of gaming software, for example, the same quality might be perceived as inadequate in aging people. In a comparative study of VR urban and natural environments, Wang et al. (42), found urban settings to be more restorative than parks, contrary to expectations. This unexpected result was attributed to the park's design, which featured bright colors and high saturation, potentially causing visual discomfort. This example highlights the importance of careful design in VR experiments, particularly when creating complex environments. Attention to design elements can enhance validity and efficacy, ensuring that intended effects are accurately achieved and evaluated. Further, when patients feel comfortable in a virtual environment (VE) that reflects their needs, users are more likely to respond naturally and authentically, providing more reliable data for assessment and improving treatment outcomes. Considering UX can also help prevent side effects (i.e., cybersickness), which can be particularly problematic considering symptoms like nausea, headache, vertigo, and blurred vision just to name a few. A well-designed UX can reduce the danger of adding further risk to the already fragile condition of patients, making VR safer and more comfortable. Moreover, fundamental aspects of the effectiveness of VR-based therapies depend on a well-designed UX, such as the sense of presence and immersion (2). Literature now concurs that these specific features collectively contribute to generating the illusion and sensation of being inside a world, even if it exists only in an artificial setting. Variables able to create this type of illusion are primarily users and media characteristics (43). On one hand, subjective attitudes may influence the sense of presence and immersion; on the other hand, these factors depend on the optimal integration of technical features and the design of VR applications (44). The aforementioned illusion, for example, is contingent upon the integration of interface characteristics, the real-time responsiveness of the environment, and the subject's perception of the environment as credible. Engagement and motivation also play a role in technology use, significantly influencing various aspects of UX (45, 46), as well as in healthcare due to the complex nature of medical procedures and the need to avoid dropping out during treatments. On one hand, user satisfaction may be markedly improved when engagement and motivation are high, fostering positive emotional connections with the technology (19). On the other hand, when users find an application as well as easy to use, also engaging, and motivating, they are more likely to use it consistently and as intended, potentially leading to better diagnostic accuracy and health outcomes.

The integration of contextual factors in UX evaluation represents a paradigm shift from traditional usability assessment toward a more comprehensive understanding of technology implementation. While context design (e.g., visual and interactive elements) remains important for creating engaging and therapeutic experiences, the success of VR interventions depends on how well these technologies integrate into the environments where they will be used.

It is evident how evaluating and optimizing UX- encompassing both user-centered design and contextual implementation factors- can help overcome such barriers to fully exploit the potential offered by VR, as well as allow for the customization of VR applications based on the specific needs of users and their environments. Depending on their traits, users are unwilling to adopt technology if they believe it does not match their wants and preferences or cannot be incorporated into their daily activities (47, 48). This user-centered approach can lead to more targeted and effective interventions, considering aspects such as accessibility, inclusivity, and safety. A prelaminary evaluation points out potential issues relayed to clinical conditions that might cause discomfort, allowing such type of adjustment. Identifying and addressing UX issues early in the development process also provides a cost-effective advantage over costly redesigns or low adoption rates after deployment.

In conclusion, investing in and improving UX appears to lead to significant advances in the diagnosis and treatment of various physiological and pathological conditions, offering a particular focus on patient care, considering a holistic perspective as the term experience underpins.

### 1.3 Objectives

Considering the potential of VR and the importance of evaluating UX, the present work aims to identify, map, and discuss the most used instruments to evaluate the experience related to VEs in healthcare, offering a comprehensive perspective on the intersection of VR technology, UX, and healthcare. Despite the diverse array of methodologies available for assessing UX, including observation, interviews, and psychophysiology approaches (49), this study focuses on questionnaires. This focus on questionnaires is justified by their practical advantages in clinical contexts, combining efficiency, ease of administration, and suitability for implementation within healthcare settings where time constraints and patient considerations are paramount (49, 50). Furthermore, this study intends to examine the principal UX dimensions that are particularly pertinent to VR experiences in healthcare, considering factors that influence technology implementation in clinical environments. Finally, the review aims to provide forward-looking insights into potential future directions in this field, offering guidance for researchers, clinicians, and technology developers seeking to optimize UX evaluation for VR applications in healthcare contexts. Through this comprehensive analysis, the work aspires to support the development of more effective, accessible, and user-centered VR.

## 2 Methods

Given our objective, this study proposed a scoping review methodology as the most appropriate approach. This methodology allows for a comprehensive exploration of the existing literature, facilitating a broad overview of the field and enabling us to synthesize diverse results, identifying and analyzing knowledge gaps (51–53).

### 2.1 Literature search

This systematic scoping review was conducted on February 2th, 2024, on three databases: PubMed, Web of Science, and Embase, and reported according to the PRISMA-ScR (Preferred Reporting Items for Systematic Reviews and Meta-Analyses, extension for Scoping Reviews) guidelines (54). Database selection prioritized comprehensive coverage while maintaining feasibility: PubMed for biomedical literature, Web of Science for multidisciplinary technology and psychology content, and Embase for European clinical research. Other databases (e.g., Scopus, IEEE Xplore) were excluded as their content was adequately captured through our approach.

We developed our search string, as shown in Table 1, through an iterative process with expert consultants and analysis of sensitivity through variations of key terms. The search string was combined as follow: (measurement OR assessment OR evaluat*) AND (“user experience” OR UX) AND (instrument OR questionnaire OR scale) AND (VR OR “virtual reality” OR “360° video*” OR “360° image*” OR “360-degree video*” OR “360-degree image*” OR “spheri* video*” OR “360° technology” OR “360-degree technology” OR “360 degree technology” OR “immersive video*” OR “immersive image*” OR “360 degree medi*” OR “virtual-based” OR virtual OR immersive OR simul*). The research strategies were narrowed based on the titles and abstracts of the records. The literature research was limited to titles and abstracts to maintain the methodological feasibility typical of scoping reviews [aiming to map the landscape of literature rather than carry out exhaustive analyses (51)], with an inclusive threshold to maximize sensitivity in the initial screening phase.

**Table 1:** The table summarizes the search string development process.

Component	Key terms	Rationale	Alternative terms tested
Measurement	Measurement OR assessment OR evaluat*	Capture all evaluative approaches including evaluation, evaluating, evaluated	measure*, assess*, apprais*, review*, analys*
User experience	“User experience” OR UX	Standard terminology in HCI field; quotation marks ensure phrase matching	“user-experience”, “customer experience”, usability
Instruments	Questionnaire OR scale	Focus on quantitative measurement tools	tool*, measure*, metric*, index*
VR technology	VR OR “virtual reality” OR “360° video*” OR “360° image*” OR “360-degree video*” OR “360-degree image*” OR “spheri* video*” OR “360° technology” OR “360-degree technology” OR “360 degree technology” OR “immersive video*” OR “immersive image*” OR “360 degree medi*” OR “virtual-based” OR virtual OR immersive OR simul*	Comprehensive coverage of VR and related immersive technologies	“mixed reality”, “extended reality”, XR, MR

### 2.2 Screening and selection

All articles retrieved from the literature search were imported into Ryyan, where duplicates were automatically removed. Two independent researchers checked the literature, following this screening process: first, titles and abstracts were analyzed. Full texts were obtained if at least one reviewer believed an article met the inclusion criteria. Subsequently, they verified eligibility through full-text screening. When conflicts arose, the researchers attempted to reach a consensus through discussion. If a resolution could not be achieved, a third researcher was consulted to arbitrate and make a final determination.

The following hierarchy of inclusion criteria was adopted for both title and abstract, and full-text screening:

1. Articles in English;
2. Experimental articles;
3. Human subject involvement;
4. Adult population;
5. Use of virtual reality or 360-degree media in healthcare;
6. Application or construction of an instrument to assess user experience (regardless of specific UX operationalization).

If articles did not respect one of the previous conditions, they were excluded for reasons corresponding to one of the following exclusion criteria:

1. Non-English articles (excluded due to resource limitations for translation and validation);
2. Review articles, meta-analyses, concept papers, and protocols (excluded as they do not provide primary empirical data on UX evaluation);
3. Studies involving only non-human subjects (excluded as UX is inherently a human-centered construct);
4. Studies focusing exclusively on children/adolescents (excluded due to our specific interest in adult population);
5. Studies not involving VR or 360-degree media in healthcare contexts (excluded as outside our research scope);
6. Studies without UX evaluation instruments (excluded as they do not address our primary research question).

### 2.3 Data extraction and synthesis

A designated researcher supervised the accuracy and completeness of the entire procedure. Completed the screening phase, resulting articles were collected, and data were extracted on Excel sheets. The following variables were extracted: population involved in the UX evaluation, VR applications (e.g., type of tasks, VEs, and technology adopted), and UX evaluation (e.g., procedures, type and features of instruments). Table 2 shows the details.

**Table 2:** The table summarizes the included studies and extracts information.

Paper	Population	VR applications	VR technology	UX assessment procedure	UX evaluation
(59)	10 healthy adults	Interactive scenes for depression assessment in VR environment composed of natural environments (e.g., green grove) and clinical scene similar to real diagnosis environment with image, language and defined gesture of characters.	HMD to deliver immersive 360° based contents	After a VR session users were asked to fill UX questionnaires.	*ad hoc* survey
(57)	45 healthy adults	Nature landscapes (grassland, forest, stream land, and beach) where users performed walk abilities. It can include a virtual trainer as a companion during the walks to increase encouragement)	HMD to deliver immersive VR contents integrated with and a platform to walk (Cardiostrong Cross trainer)	Participants performed three consecutive days of intervention at the end they performed Ux questionnaire.	UEQ
(58)	40 healthy adults	3 different game in which participants have to performd several occupations, such as a cook, car mechanic, and an office worker (Job Simulator), complete several mini-games like slingshot, or longbow (The Lab), and complete several imaginary home chores (Rick and Morty).	HMD and motion sensors to deliver immersive VR contents	Participants performed 3 sessions of intervention (one per week), then they performed UX questionnaires.	Virtual Reality Neuroscience Questionnaire (VRNQ)
(62)	23 healthy adults	Users performed three exergame: (i) wall dodging (players must rapidly maneuver through holes in approaching walls); (ii) fruits picking (three fruits are randomly displayed on the screen and users should try to catch a specified fruit by moving the body from side to side); (iii) rats stomping (participants score points by stepping on rats that emerge from the holes).	Motion interaction sensors connected with a screen displaing sound feedback and motion interaction.	After a VR session users were asked to fill UX questionnaires.	User Experience Questionnaire (UEQ-S) short version
(61)	66 healthy adults	EXIT 360°: domestic photos as virtual environments in which participants have to perform seven subtasks of increasing complexity (e.g., observe a map and choose the right path to exit to the house, explore a room and select the correct person according to a specific instruction, solvea rebus, memorize a sequence of numbers and report them in reverse)	HMD to deliver immersive 360° based contents	After a VR session users were asked to fill UX questionnaires.	User Experiece Questionnaire; ICT—SOPI; Flow Short Scale (three items); Intrinsic Motivation Inventory (subscale enjoyment—four items)
(69)	27 PD patients and 27 healthy adults	EXIT 360°: domestic photos as virtual environments in which participants have to perform seven subtasks of increasing complexity (e.g., observe a map and choose the right path to exit to the house, explore a room and select the correct person according to a specific instruction, solvea rebus, memorize a sequence of numbers and report them in reverse)	HMD to deliver immersive 360° based contents	After a VR session users were asked to fill UX questionnaires.	User Experiece Questionnaire; ICT—SOPI; Flow Short Scale (three items); Intrinsic Motivation Inventory (subscale enjoyment—four items)
(68)	15 healthy adults and 7 stroke patients	Natural outdoor scene (vegetable garden, lake and forest) presented in one of three lighting conditions (day, evening or night). Users have performed visual discrimination tasks.	HMD to deliver immersive VR contents	In a pre-training phase, cybersickness symptoms was measured. After a VR session users were asked to fill all the UX questionnaires.	SSQ *ad hoc* User Experience scale
(64)	15 MCI patients	Virtual supermarket filled with grocery items, and the cash-register scene, in which the users can pay for the items they had picked. Users had shopping, picking all the items presented on a list and putting them in a cart.	HMD to deliver immersive VR contents	In a pre-training phase cybersickness symptoms and and intention to use the virtual reality system were measured. After a VR session users were asked to fill all the UX questionnaires.	Simulator Sickness Questionnaire (SSQ); International Test Commission—Sense of Presence Inventory (ITC-SOPI); Technology Acceptance Model 3 questionnaire
(55)	20 healthy adults	360° natural environments characterized by auditory stimuli. Users may choose one of the preoposed realistic scenario (mountain, marine, and countryside environment) where experience relaxation training.	HMD to deliver immersive 360° based contents	After a VR session users were asked to fill UX questionnaires.	*ad hoc* questionnaire
(60)	23 healthy older adults and healthcare experts	15 games from GAME2AWE platform organized into two themes (Life on a Farm and Fun Park Tour). Activiteies are themed around farming (e.g., seeding and fertilizing a field, crop harvesting, insect repelling, and selling crops or purchasing resources) and in fun park (activities that require physical and cognitive skills) respectively.	GAME2AWE platform composed of movement traking sensors and HMD to deliver immersive VR contents	UX measure were assessed in a pre pilot phase from experts and final users; and in a pilot phase from end-users.	Short interviews and discussions with seniors and experts; System Usability Scale (SUS) questionnaire; Virtual Reality Sickness Questionnaire (VRSQ); Acceptance and Use of Technology (UTAUT) model questionnaire
(65)	30 elderly patients with post-stroke cognitive impairment	16 games grouped in 3 categories in wich users performed life skills training (cooking, cleaning a window, crossing a road, watering flowers), exergames (playing sqash, shooting antiaircraft guns, flying gliders, playing baseball) and entertaining games (bracking eggshells, swatting insects, lighting fireworks, whack a mole, pumpong un a ballon, flying a Kongming lantern, Fruit Ninja, bubble jab).	HMD and motion sensors to deliver immersive VR contents	Participants were involved in 6 weeks of training, then UX was evaluated.	self-reported questionnaire
(63)	35 patients with brain injury resulting in attention deficits	VR traveller: attentional dysfunctions program composed of several modules, within the context of a virtual journey around the world.	HMD to deliver immersive VR contents	After the testing modules (one time) participants filled out the questionnaires and were interviewed about their experience.	User Experience Questionnaire (UEQ)
(29)	60 college women	A room without any furniture except for a large mirror located in front of the participant and two boxes placed on the floor beside users. They see their whole image in the mirror (avatar) and perfor an attentional bias modification task procedure.	HMD and motion sensors to deliver immersive VR contents.	After a VR session users were asked to fill UX questionnaires.	System Usability Scale (SUS)
(70)	23 healthy adults and 22 with lower limb disorder	Pedaleo VR: three scarios (sky, canyon valley and sailing environment) in which participants had to control a vehicle by pedaling. Vehicles might be a light aircraft or a fishing vessel.	cycle-ergometer and HMD to deliver immersive 360° based contents	After a VR session users were asked to fill UX questionnaires.	Intrinsic motivation inventory (three subscales); Credibility and expectancy questionnaire (CEQ); Simulator sickness questionnaire (SSQ); Presence questionnaire (PQ); 18-item short scale of Game user experience satisfaction scale (GUESS); System usability scale (SUS)
(67)	21 ADHD patients and 21 neurotypical participants	Zenctuary VR: small garden surrounded by a forest and a gently flowing river. Users had to pleased in the virtual space, interact with the environment as they wanted (various types of auditory, visual and tactile responses to the users’ actions were generated), or even just look around the garden populed with flowers, birds, cloud, batterflies, plants.	HMD to deliver immersive VR contents	After a VR session users were asked to fill UX questionnaires.	User eXperience in Immersive Virtual Environment questionnaire (UEIVE)
(66)	14 patients with COPD	Virtual Park: park with graphical and audio elements typical of a natural environment (e.g., flowers, trees, birds) where users simulate a bicycle ride.	Cycle-ergometer and and a wide projected screen in front of the bicycle to provide semi-immersive VR experience.	Participants were involved in 2 weeks of training, then UX was evaluated.	Modified version of the User Experience Questionnaire (UEQ); Short Flow State Scale 2 (SFSS-2)
(56)	15 healthy adults	Hospital scenario simulating the experience of a morally challenging event related to the COVID-19 pandemic.	HMD to deliver immersive VR contents	After a VR session users were asked to fill UX questionnaires.	Igroup Presence Questionnaire (IPQ); dropout rate; qualitative responses provided during the debrief

## 3 Results

**Figure 1:**
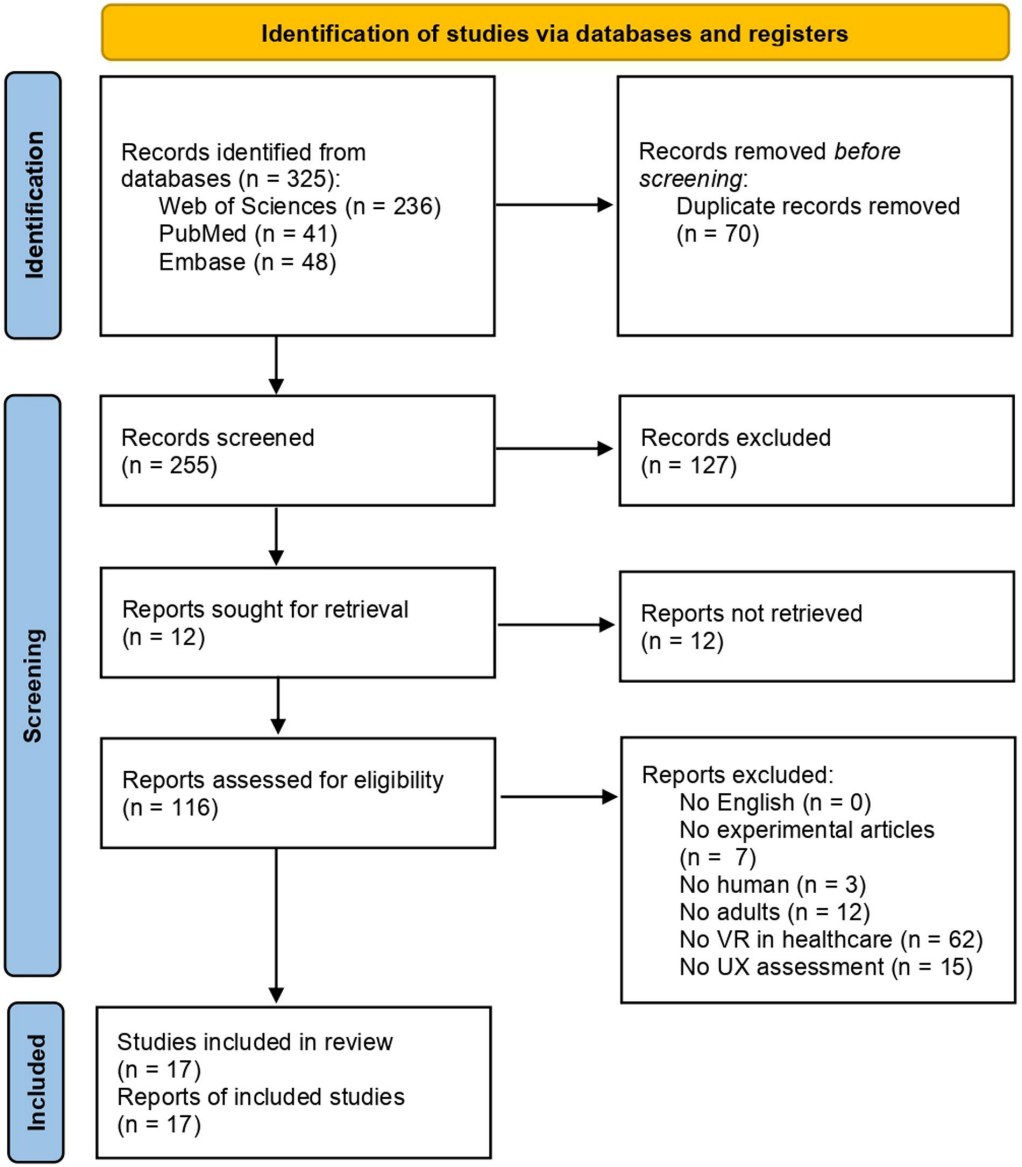
PRISMA flowchart of the included studies.

Seventeen articles resulted from the described literature review process. The general process was described using a flowchart as shown in Figure 1.

### 3.1 Population

Most of the studies involved non-clinical populations. In particular, Pardini and colleagues (29, 55–58) recruited young adults with a mean age of 34.2 ± 10.6, 24.83 ± 6.64, 32.7 ± 9.5, 21.82 ± 1.84, and 32.08 ± 3.54 respectively. The study of Liao et al. (59) involves subjects between 21 and 37 years old. Goumopoulos and colleagues (60) recruited two groups of healthy adults (mean age 71.3 ± 4.3 and 67.5 ± 5.8 respectively) and healthcare experts from different fields of medicine such as physiotherapy, orthopedics, psychology, physical education, and sports science. Borgis et al. (61) and Chen et al. (62) involved healthy older adults with a mean age of 53.5 ± 20.30, and 71.48 ± 4.09 years respectively.

Eight studies recruited adults with different diseases. Lorentz and collaborators (63) test their intervention on patients with brain injury resulting in attention deficits (mean age 51.66 ± 17.8). Patients with Mild Cognitive Impairment (MCI) were actors in one study (64) (mean age 75.73 ± 6.36). Similarly, patients with post-stroke cognitive impairment were recruited by Liu et al. (65) (mean age 74.93 ± 6.81). Colombo and colleagues (66) recruited patients with mild/moderate COPD (Chronic Obstructive Pulmonary Disease) (mean age 71.29 ± 6.93). Tölgyesi and collaborators (67) involved ADHD patients (mean age 31.9 ± 8.8) compared with neurotypical participants (mean age 34.4 ± 8.9). Huygelier et al. (68) compared stroke patients (mean age 44 ± 19) with neurological healthy controls (mean age 59.57 ± 9.78). Patients with Parkinson's Disease (PD) were involved in one study (mean age 68.2 ± 9) compared with healthy controls (mean age 66.4 ± 10.5) (69). Rojo and colleagues (70) tested their application on older adults and patients with lower limb disorder, with a mean age of 85.16 ± 5.93 and 61.10 ± 12.62 respectively.

### 3.2 VR applications

Most of the examined studies can be classified as preliminary investigations focusing on feasibility and acceptability. These studies aimed to evaluate the viability of proposed interventions, focusing on aspects related to the experience of users in interacting with VR applications or specific components thereof.

Most applications (*n* = 15) utilized computer-generated virtual environments, while a smaller subset (*n* = 4) employed 360° media capturing real-world environments. The predominance of HMD-based delivery systems (*n* = 17) reflects the current technological preferences in VR healthcare research, with additional hardware integration including motion sensors, exercise equipment, and projection systems depending on the specific application requirements.

The interventions predominantly target to train various aspects such as cognitive and motor functions. These studies represented the largest category, encompassing applications designed to train attentional abilities, executive functions, and motor skills through interactive tasks. These applications typically used computer-generated environments simulating real-life settings such as cities or part of the world, gardens, lakes, canyon valley, and seas. They predominantly employed Head-Mounted Displays (HMDs) to deliver structured training protocols (60, 63, 65, 68, 70), with some incorporating motion sensors integrated with VR displays (29, 62) to track body movements, or combining cycle-ergometers with wide projected screens positioned in front of bicycles (66) to improve motor performance in a park. Other applications focused on therapeutic, and wellness aimed at stress reduction, anxiety management, and general well-being, often featuring natural environments such as gardens, parks, or scenic landscapes. These applications emphasized relaxation and emotional regulation rather than specific skill training (55, 57, 67). All studies in this category use HMDs for content delivery.

Four applications specifically recreated real-world scenarios for training purposes, including shopping environments (64), hospital situations (56), and domestic tasks (61, 69). These applications aim to provide safe practice opportunities for complex real-world activities, allowing users to develop skills in controlled virtual settings before applying them in actual contexts.

One study specifically aims at designing personalized virtual environments using 360° content to detect depression (59), and another focused on validity examination of a questionnaire to assess the quality of the VR experience (58). These studies highlight the ongoing development of both therapeutic applications and measurement tools in the field.

### 3.3 UX evaluation

Most studies interviewed participants about their experience immediately after the VR sessions and they were mainly required to fill out one or more questionnaires (29, 55, 57–63, 65–67, 69, 70). Conversely, (64), as well as (68), measured cybersickness symptoms both before and after the intervention, in addition to some other UX scale after the VR experience.

Four studies (57, 62, 63, 66) used the User Experience Questionnaire (UEQ) (71), in its original (57, 63), short (62) or modified by authors (66) version. Colombo and colleagues (66) add to the UEQ the Short Flow State Scale 2 (SFSS-2) (72).

Four studies provided *ad hoc* questionnaires. Particularly (59), created a survey to indicate the user's satisfaction in a range from 1 (dissatisfaction) to 5 (satisfaction). (55) take cues from 8 items from the Virtual Reality Symptom Questionnaire (VRSQ) by (73) and 37 items from the Presence Questionnaire and the Immersive Tendencies Questionnaire (74). (65) also designed their self-reported questionnaire composed of 14 items divided into three parts: frequency of smart device usage; satisfaction; occurrence and degree of adverse reactions during the intervention. (68) also design an *ad hoc* UX scale based on the International Test Commission—Sense of Presence Inventory (ITC-SOPI) (75), Narrative Engagement Scale (76), and intrinsic motivation inventory (77). They also administered the Simulator Sickness Questionnaire (SSQ) (78). All these studies used closed-ended responses (Likert scales).

(67) evaluated UX through the User eXperience in Immersive Virtual Environment questionnaire (UEIVE) (79). (64) used the SSQ (78), ITC-SOPI (75), and the Technology Acceptance Model 3 questionnaire (80). (56) proposed the Igroup Presence Questionnaire (IPQ) (81); moreover, the authors evaluated the dropout rate and qualitative responses (i.e., content analysis) provided during the debrief to assess UX. (60) evaluated experts' and users' opinions during the design phase of the VR application, through short interviews and discussions; then the authors required participants to fill out the System Usability Scale (SUS) questionnaire (82), the VRSQ (73), and the Acceptance and Use of Technology (UTAUT) model questionnaire (80). SUS was also used by (29). (61, 69) presented the UEQ (71), ICT-SOPI (75); three items from the Flow Short Scale (83), and four items from the Intrinsic Motivation Inventory (subscale enjoyment) (84). (70) used the Intrinsic Motivation Inventory (84), Credibility and Expectancy Questionnaire (CEQ) (85), the SSQ (78), Presence questionnaire (PQ) (74), SUS (82), and the 18-item short scale of the Game User Experience Satisfaction Scale (GUESS) (86). Finally, the Virtual Reality Neuroscience Questionnaire (VRNQ) was designed by (58). Table 3 shows details of the questionnaires and relative variables.

**Table 3:** The table displays the instruments each study employed, and the specific variables chosen by the authors to assess UX.

Paper	UX evaluation	Variables involved in UX evaluation
(59)	*ad hoc* survey	Easy to learn, interest, complexity, attractiveness, naturalness, definition, visibility, vertigo
(57)	User Experience Questionnaire (UEQ)	Attractiveness, efficiency, perspicuity, dependability, stimulation, novelty
(58)	Virtual Reality Neuroscience Questionnaire (VRNQ)	User experience (intensity of the immersion, the level of enjoyment, quality of the graphics, sound, and VR technology), Game mechanics, In-game assistance, VR symptoms and effects
(62)	User Experience Questionnaire short version (UEQ-S)	Pragmatic and hedonic quality
(61, 69)	User Experience Questionnaire (UEQ)	Attractiveness, efficiency, perspicuity, dependability, stimulation, novelty
	International Test Commission -Sense of Presence Inventory (ITC-SOPI)	Spatial presence, engagement, naturalness, side-effects
	Flow Short Scale (three items)	Abilities in coping with the task, challenges, challenge-skill balance
	Intrinsic Motivation Inventory (four items)	Enjoyment
(68)	Simulator sickness questionnaire (SSQ)	Nausea, oculomotor disorders, disorientation
	*ad hoc* User Experience scale	Usability of the touch controllers and user interface, Amount of presence experienced, Experience of the narrative, Motivation
(64)	Simulator Sickness Questionnaire (SSQ)	Nausea, oculomotor disorders, and disorientation
	International Test Commission -Sense of Presence Inventory (ITC-SOPI)	Spatial presence, engagement, naturalness, side-effects
	Technology Acceptance Model 3 questionnaire	Perceived ease of use, computer anxiety, perceived enjoyment, behavioral intention
(55)	*ad hoc* questionnaire	General physical side effects (e.g., fatigue, headache, nausea, concentration difficulties), Visual effects (e.g., blurred vision, and tired eyes), Realism of the environments, engagement, Immersiveness, tools’ Usability and quality of the interface, Emotional states, Satisfaction
(60)	Short interviews and discussions with seniors and experts	Whether such games could have a positive impact and to identify any features that must be implemented in the future
	System Usability Scale (SUS) questionnaire	Effectiveness, efficiency, satisfaction
	Virtual Reality Sickness Questionnaire (VRSQ)	General discomfort, fatigue, eyestrain, difficulty focusing, headache, fullness of head, blurred vision, dizzy, vertigo
	Acceptance and Use of Technology (UTAUT) model questionnaire	Performance expectancy, Effort expectancy, Social Influence, Facilitating conditions
(65)	Self-reported questionnaire	3 parts: (i) how often individuals used smart devices before the intervention, (ii) satisfaction with equipment and training content, (iii) occurrence and degree of adverse reactions during the intervention
(63)	User Experience Questionnaire (UEQ)	Attractiveness, efficiency, perspicuity, dependability, stimulation, novelty
(29)	System Usability Scale (SUS) questionnaire	Effectiveness, efficiency, satisfaction
(70)	Intrinsic motivation inventory (three subscales)	Value/usefulness, interest/enjoyment, perceived choice
	Credibility and Expectancy Questionnaire (CEQ)	Credibility and expectancy
	Simulator sickness questionnaire (SSQ)	Nausea, oculomotor disorders, disorientation
	Presence questionnaire (PQ)	Realism, control, quality of interface, possibility to examine, possibility to act, and self-evaluation.
	18-item short scale of Game user experience satisfaction scale (GUESS)	Usability/playability, narratives, play engrossment, enjoyment, creative freedom, audio aesthetics, personal gratification, social connectivity, visual aesthetics
	System Usability Scale (SUS)	Effectiveness, efficiency, satisfaction
(67)	User eXperience in Immersive Virtual Environment Questionnaire (UEIVE)	Presence, engagement, immersione, flow, skill, emotion, usability, technology adoption, judgment, experience consequence
(66)	Modified version of the User Experience Questionnaire (UEQ)	Attractiveness, perspicuity, stimulation, novelty
	Short Flow State Scale 2 (SFSS-2)	Challenge–skill balance, action–awareness, clear goals, unambiguous feedback, Concentration on the task at hand, sense of control, transformation of time, loss of self-consciousness, autotelic experience
(56)	Igroup Presence Questionnaire (IPQ); dropout rate; qualitative responses provided during the debrief	General presence, spatial presence, involvement, experienced realism

Results reveal that authors assessed self-declared UX measures rather than theoretically consistent constructs, with most studies presenting instruments aimed at evaluating isolated application features. For instance, some studies have reduced UX evaluation to purely usability-focused measures, limiting their scope to technical and functional aspects. However, UX represents a multidimensional construct that integrates traditional concepts while extending beyond them to encompass broader dimensions. Rather than replacing previous frameworks, UX builds upon established constructs such as usability, incorporating them within a more comprehensive understanding of human-technology interaction. From this perspective, we sought to provide a comprehensive overview of UX aspects based on the identification of the most frequently assessed dimensions in VR literature. We analyzed and cataloged all variables from the examined questionnaires, identifying which variables were most commonly employed by authors across all reviewed studies. Variables that were semantically similar were grouped together into coherent clusters through expert consensus. Our analysis revealed that the most frequently utilized variables in the literature clustered into eight distinct groups, thereby establishing our 8-factor model that represents the variables most commonly used to evaluate UX in VR contexts. The eight key UX factors emerged from our analysis:

*Usability and functionality* focus on the technical aspects of VR applications. This domain encompasses variables such as efficiency (the product's ability to enable quick and optimal use), perspicuity (clarity and comprehensibility of the interface), dependability (i.e., how confident and in control the user feels when using the product, it refers to the reliability and predictability of the system), effectiveness (how well a system enables users to complete specific tasks), easy to learn and use.
i. A*esthetics of design* considers the visual and creative aspects of the VR environment, including attractiveness (general aesthetics and appeal) and design aspects such as innovation, creativity, and cutting-edge (novelty) (23, 87).
ii. *Engagement*, involving variables that capture the user's active participation and involvement in the VR experience, including stimulation (whether the product captures the user's attention and engages participants), the degree to which users are mentally and emotionally involved in the experience (involvement), immersion, and flow states (88).
iii. *Emotional state* encompasses the affective responses elicited by the VR experience, including both positive and negative feelings.
iv. *Presence* represents a core psychological construct in VR research, referring to the subjective experience of being in one environment when physically situated elsewhere (74, 89). This construct captures the user's sense of “being there” in the virtual space and is fundamental to successful VR experiences.
v. *Realism of the environments* refers to the degree of resemblance between virtual environments and their real-world counterparts. While related to presence, realism specifically addresses the fidelity and authenticity of virtual representations, including visual, auditory, and behavioral similarities to reality. This factor is conceptually separate from presence as users can experience high presence in fantastical, unrealistic environments, while realistic environments may not necessarily evoke strong presence sensations (90, 91).
vi. *Side effects* including general physical side effects (e.g., nausea, vertigo, fatigue, headache, disorientation) and visual side effects (e.g., oculomotor disorders, eyestrain, blurry vision) that may negatively impact the user experience (78, 92).
vii. *Motivation and intention of use* encompasses the psychological drivers and behavioral inclinations that influence user engagement with VR technology. Motivation represents the underlying forces that energize and direct user behavior within the VR environment, while intention of use captures the user's willingness and planned commitment to engage with the technology. From a cognitive science perspective, intention involves hierarchical representations of future desired states that guide user actions within the VR environment. From a technology acceptance perspective, based on the theory of planned behavior, behavioral intention represents the user's willingness to engage with the technology. This factor also includes motivation (the driving forces behind user behavior), perceived usefulness, and technology adoption patterns (89, 93, 94).

**Figure 2:**
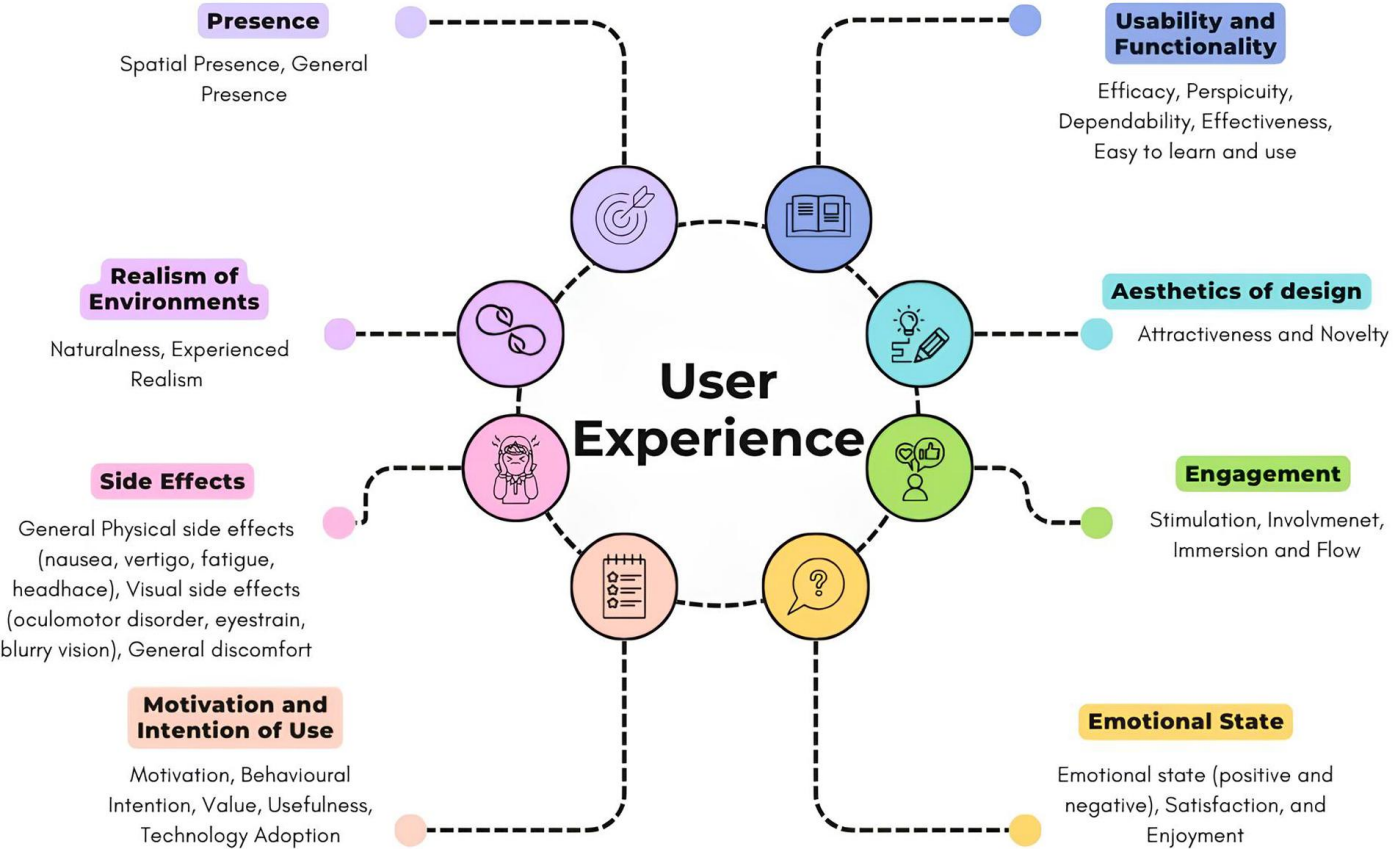
Summarization of the key essential factors, each representing a cluster of variables derived from most used UX questionnaire.

Figure 2 illustrates the groups of variables into the mentioned possible key factors.

## 4 Discussion

This review examines the cutting-edge questionnaires currently utilized for evaluating UX in VR applications for healthcare. Our analysis encompasses some key aspects such as the target population of UX studies, the proposed VR applications, and the instruments employed, including their respective dimensions, as considered by researchers to assess UX comprehensively. We observed that (i) while the majority of participants were healthy adults, a substantial number of studies also included clinical populations, particularly individuals with neurological conditions; (ii) VR applications involved in UX evaluation predominantly focused on training cognitive and motor abilities, and they featured immersive, interactive, and computer-generated environments; (iii) UX evaluation typically occurred immediately following the VR experience; (iv) researchers employed multiple questionnaires to capture a comprehensive range of experiential aspects; (v) we noted that eight key domains emerged as the most frequently assessed variables in UX studies.

During the last decades, significant research efforts have been directed towards innovative instruments to promote healthcare, help people deal with their medical conditions, and support the well-being of several patients and their caregivers. The UX has been identified as a key aspect in designing and implementing these instruments. The quality and design of instruments significantly influence whether they achieve widespread acceptance and clinical success or fail to gain traction in healthcare settings (95). Thus, required users' feedback is crucial, and it is essential to conduct studies directly involving end-user populations, whether patients or health people. Through this direct engagement, clinicians and developers can gain comprehensive insights into the authentic needs, preferences, and challenges associated with implementing these applications in clinical settings. However, an often neglected—yet equally significant—aspect is the incorporation of UX expert opinion in the assessment process. Notably, our results revealed a striking lack in this regard: only a single study explicitly reported expert feedback during the preliminary design phase. Moreover, the inclusion of multidisciplinary figures could be useful in the UX process. Specialists, therapists, and health researchers might provide valuable insights based on their clinical and technical experience. Their opinion can be instrumental in identifying potential usability issues, assessing the accuracy and clinical relevance of content, and suggesting improvements that may be missed by end users or designers, offering a more comprehensive evaluation framework. Integrating end-user and expert perspectives facilitates a holistic approach to UX assessment. This multifaceted evaluation strategy reflects long-term clinical efficacy, safety considerations, and integration of healthcare systems. By synthesizing diverse viewpoints, it may be possible to create VR applications that are engaging, user-friendly, clinically effective, and suitable for real-world healthcare settings.

UX evaluation focuses on the immediate effects of interaction. Notably, all extant studies in this work have employed real-time data collection methods to capture user responses promptly upon completion of the VR intervention. However, this approach is not universally applicable, as there are instances where the effects of the experience may be confounded with baseline conditions, and vice versa. This is the case of adverse physiological reactions, such as headache, nausea, and vertigo. While these symptoms may be attributable to the side effects of the VR application, it is also plausible that participants may have experienced these conditions before the VR exposure, thus potentially skewing the results. To address this methodological issue, specific psychometric instruments have been developed to capture these nuanced aspects of experience, such as the SSQ (78). This tool measured cybersickness symptoms pre- and post-intervention, enabling researchers to evaluate potential changes attributable to the experimental condition. Moreover, a critical—yet often overlooked aspect—is the long-term effect of interventions. While analyzed studies focus on single-session outcomes, it would be important to investigate whether effects persist over time or across multiple sessions (66). This longitudinal perspective is particularly crucial when considering patient populations, such as chronic conditions. Patients might exhibit reduced exercise tolerance and diminished motivation in activities for example. These factors can significantly impact their engagement with interventions.

Despite these considerations, many questionnaires were designed over time to evaluate UX. However, the measurement tools reported in this review predominantly reflect authors' self-declared UX measures rather than theoretically validated UX constructs, thereby highlighting the field's current fragmentation and lack of consensus regarding what constitutes UX. This methodological inconsistency is exemplified by studies that merely capture usability measures using instruments such as the SUS rather than assessing the multidimensional nature of user experience. This approach results in a proliferation of disparate questionnaires, some of which fail to capture the multifaceted nature of UX, risking the reduction of this complex phenomenon to disparate constructs that are merely grouped under the UX umbrella.

A critical examination of proposed instruments reveals several fundamental limitations A significant proportion of tools are outdated (e.g., 72, 77, 82), and may not fully encompass the complexities of modern technologies. Furthermore, many extant instruments were originally developed for traditional computer interfaces or conventional media context such as the SUS (82), the ITC-SOPI (75), yet have been frequently employed across diverse research contexts without adequate adaptation. The appropriateness of these instruments for evaluating novel interaction methods in VR environments is questionable, given that their original design parameters did not consider the specific dimensions that characterize VR contexts, such as immersion, presence, and spatial interaction. Moreover, a notable paucity of cross-cultural validation exists for many of these questionnaires, particularly in non-English speaking contexts. In most cases, tools were merely translated into a different language, causing possible validity issues when conducting rigorous UX evaluations. The absence of an appropriate validity evaluation of tools may compromise the accuracy of UX measurements across different contexts and the reliability and validity of results, inadequately capturing UX elements. For instance, in the Italian research landscape, there is a conspicuous absence of tools, to the best of our knowledge (75, 82).

To address this gaps, researchers frequently employ multiple questionnaires in an attempt to encompass the myriad of objective and subjective variables. While this approach facilitates a more comprehensive assessment, it also introduces challenges related to respondent burden and potential construct overlap., and lack of theoretical framework guiding instrument selection. Through our systematic analysis of the literature, we identified a substantial convergence of variables that we organized into eight key theoretical domains based on their distinctive characteristics. These domains encompass a wide range of aspects crucial to the UX in VR, such as usability and functionality, aesthetics of design, engagement, emotional state, presence, realism and naturalness of the environments, side effects, and motivation and intention of use. This eight-factor theoretical framework represents a comprehensive model for UX evaluation in VR-based healthcare applications, derived from empirical evidence across the reviewed studies.

## 5 Conclusion

Given the discussed significance of considering UX in the development of VR applications, this review has presented an analysis of the most frequently utilized instruments in healthcare settings. Through a synthesis of the evidence presented herein, and by integrating insights from the broader corpus of literature, we propose several key recommendations in the design and suitability analysis of VR tools. These proposals aim to enhance the methodological rigor and efficacy of UX evaluation questionnaire-based in healthcare VR applications:

1. Integration of multidisciplinary expertise: collaboration between a diverse team of experts and end-users ensures a holistic perspective on the applications' impact in real-world healthcare settings. This multidisciplinary approach might encompass specialists in human-computer interaction and VR-specific design principles, technical experts, and domain-specific clinicians (e.g., physiotherapists for physical training, psychologists for mental health interventions), to provide specialized insights into user needs and expectations.
2. Consider long-term evaluation: while immediate analysis is essential, longitudinal UX evaluation allows researchers to capture the evolution of users' perceptions, skills, and attitudes over time. This approach reveals adoption patterns and identifies potential issues that may emerge with prolonged use, which is particularly important in healthcare settings where user engagement and adherence are critical.
3. Comprehensive assessment framework: developing instruments within a holistic approach that encompass various dimensions of UX may be appreciable. This strategy should aim to combine multiple, often subjective questionnaires into a more cohesive evaluation tool. Such an approach streamlines the UX assessment process, enhancing its replicability and facilitating meaningful comparisons across studies and applications.
4. Verification of psychometric properties: rigorous psychometric evaluation, considering reliability, validity, and cultural appropriateness of instruments ensures the generalizability of results and their applicability across various cultural settings, thereby enhancing the global relevance and impact of UX evaluations in healthcare VR applications.
